# Lipidomic Perturbations in Cynomolgus Monkeys are Regulated by Thyroid Stimulating Hormone

**DOI:** 10.3389/fmolb.2021.640387

**Published:** 2021-03-15

**Authors:** Tao Xu, Yanling Yang, Xing Huang, Jianhong Ren, Ting Xu, Wei Xie

**Affiliations:** ^1^ The Key Laboratory of Developmental Genes and Human Disease, School of Life Sciences and Technology, Southeast University, Nanjing, China; ^2^ The Therapeutic Antibody Research Center of SEU-Alphamab, Southeast University, Nanjing, China; ^3^ School of Pharmacy, Yantai University, Yantai, China; ^4^ Zhejiang Provincial Key Laboratory of Pancreatic Disease, The First Affiliated Hospital, School of Medicine, Zhejiang University, Hangzhou, China; ^5^ Department of Hepatobiliary and Pancreatic Surgery, The First Affiliated Hospital, School of Medicine, Zhejiang University, Hangzhou, China; ^6^ Suzhou Bionovogene Metabolomics Platform, Jiangsu, China

**Keywords:** thyroid-stimulating hormone, recombinant human thyroid-stimulating hormone, cynomolgus monkeys, lipids, lipidomics

## Abstract

Thyroid disease affects an estimated 200 million people worldwide, and is commonly associated with increased blood lipid levels. However, the mechanism by which thyroid-stimulating hormone (TSH) affects lipid profiles is not clear. Twenty-four cynomolgus monkeys were treated with a novel exogenous recombinant human TSH (rhTSH) (SNA001) at 9 μg kg^−1^, 22 μg kg^−1^, or 54 μg kg^−1^, and reference rhTSH (Thyrogen^®^) at 22 μg kg^−1^. The primary TSH (SNA001) pharmacokinetic (PK) parameters increased in a dose-dependent manner across the dose range of 9 μg kg^−1^, 22 μg kg^−1^, or 54 μg kg^−1^. Peak triiodothyronine (T3) and thyroxine (T4) levels were reached within 24 h after rhTSH administration, which was delayed by approximately 20 h. In total, 420 lipid species were detected and quantified by ultra-performance liquid chromatography high resolution spectrometry (UPLC-HR-MS)-based lipidomics. Notably, peak levels of lipid accumulation, particularly sphingomyelin (SM) and triglycerides (TG), appeared at 4 and 24 h, which was consistent with the pattern of TSH and T3/T4 levels, respectively. According to weighted correlation network analysis (WGCNA), perturbations of many lipid species were strongly correlated with TSH and T3/T4 levels. TSH and the stimulated T3/T4 levels and lipid profiles following SNA001 administration were comparable to those after administration of the reference rhTSH (Thyrogen^®^). The plasma lipidome and changes in lipid levels after rhTSH stimulation were associated with TSH and T3/T4 concentrations. T3/T4 and lipid profiles were delayed after TSH stimulation. Such phenomena require further exploration.

## Introduction

Thyroid disease affects an estimated 200 million people worldwide (The Lancet Diabetes and Endocrinology, 2013). Hypothyroidism and hyperthyroidism are the most common thyroid disease. Hypothyroidism is defined as high TSH concentrations and low T4 levels, while hyperthyroidism is characterized by the opposite pattern with low TSH and high T4 (Taylor et al., 2018). Studies have revealed that the observed lipid abnormalities, including dysregulation of total cholesterol (TC), low-density lipoprotein cholesterol (LDL-C), triglycerides (TGs), and high-density lipoprotein cholesterol (HDL-C), were associated with the changes in the thyroid hormone levels (Rizos et al., 2011). Overt hypothyroid patients showed elevated total and low-density lipoprotein (LDL) cholesterol and triglyceride (TG) levels, while overt hyperthyroid patients showed decreased lipid levels (Peppa et al., 2011). Lipid abnormalities are observed in many thyroid diseases, such as overt/subclinical hypothyroidism and hyperthyroidism, and increase the risk of endothelial dysfunction, hypertension and cardiovascular disease (Duntas, 2002; Jabbar et al., 2017; Razvi et al., 2018).

Thyroid-stimulating hormone (TSH) is a glycoprotein produced in the pituitary gland, and targets specific receptors on thyroid follicular cells. TSH stimulates the thyroid to produce T3 and T4 (Yen, 2001). TSH, T3, and T4 also play an important role in regulating lipid metabolism (Duntas, 2002). The potential mechanisms by which TSH and thyroid hormones regulate lipid metabolism include modulation of the expression of genes, such as 3-hydroxy-3-methyl-glutaryl-CoA reductase (HMG-CoA) in cholesterol biosynthesis (Simonet and Ness, 1988), apolipoprotein AV (ApoAv) in TG regulation (Prieur et al., 2005), LDL receptor (LDLR) (Shin and Osborne, 2003) and cholesteryl ester transfer protein (CETP) (Lagrost, 1994) in LDL and high density lipoprotein (HDL) metabolism.

Although the link between thyroid diseases and serum cholesterol was well established about 90 years ago (Robert et al., 1930), measurement of lipids, including HDL-cholesterol and LDL-cholesterol, TG and TC, provide only a narrow view of lipid metabolism. The state of art UPLC-HR-MS based lipidomic techniques is a newly emerging approach that allows comprehensive analysis of large-scale analysis of hundreds to thousands of lipid species in complex biological samples (Hyotylainen and Oresic, 2015; Lydic and Goo, 2018). Lipidomics offers a new opportunity to decipher the relationships between TSH, T3, T4 and lipid profiles (Lv et al., 2018).

A newly developed recombinant human thyroid-stimulating hormone (rhTSH) SNA001 (Suzhou SmartNuclide biotech Co., Ltd), which is a heterodimeric glycoprotein expressed in Chinese hamster ovary (CHO) cells, was then employed in this study. SNA001 has an identical amino acid sequence and a similar glycosylation pattern to the human thyrotropin and the biological reference rhTSH (Thyrogen^®^, Sanofi Genzyme).

In the present study, the pharmacokinetic (PK), pharmacodynamic (PD), and lipid profiles using UPLC-HR-MS technology were assessed to identify the impacts of a single intramuscular injection of SNA001 and Thyrogen at low, intermediate and high doses in male and female cynomolgus monkeys. To the best of our knowledge, this is the first integrated study of PK/PD and lipidomic profiles of rhTSH in cynomolgus monkeys. The information obtained from this study may provide a more comprehensive understanding of the relationships among TSH, thyroid hormones and lipid profiles.

## Materials and Methods

### rhTSH

SNA001 was a heterodimeric glycoprotein expressed manufactured in Chinese hamster ovary (CHO) cells and purified by Suzhou SmartNuclide Biotech Co., Ltd. (Suzhou, China). The biological reference rhTSH (Thyrogen^®^) was purchased from Genzyme Sanofi (Cambridge, Massachusetts, United States).

### Animal Source and Care

Male and female (12 of each) naïve cynomolgus monkeys, aged 3–5 years and weighing 2.5–3.37 kg, were obtained from Guangdong Frontier Biological Technology Co., Ltd. The animals were maintained in a facility approved by the Association for Assessment and Accreditation of Laboratory Animal Care (AAALAC)-International. All experiments using animals were conducted in accordance with the principles laid out by the ethics committee of Joinn Laboratories (China) Co., Ltd. (IACUC No. ACU17-819).

### Animal Studies

A single-dose cynomolgus monkey PK study was performed using an intramuscular injection of rhTSH in the buttocks. Naïve monkeys were assigned to four groups (*n* = 6 per group; 3 of each sex): SNA001 9 μg kg^−1^, SNA001 22 μg kg^−1^, SNA001 54 μg kg^−1^, and Thyrogen 22 μg kg^−1^. A full PK profile was obtained at the following time-points: pre-test, and 0.5, 1, 2, 4, 8, 12, 24, 36, 48, 72, and 96 h post-dose. Serum T3 and T4 profiles were obtained at pre-test, 4, 8, 12, 24, 48, 72, 96, and 120 h post-dose. As a control, plasma samples were also collected at 0, 1, 2, 4, 8, 24 h in another six Naïve monkeys (3 of each sex).

### Sample Measurement

Analyses of plasma TSH levels were performed at Joinn Laboratories (China) Co., Ltd. in Suzhou. The validated chemiluminescence immunoassay (CLIA) method, with a linear range of 0.5–500 ng/ml, was used to determine blood concentrations of TSH. Total T3 and total T4 were measured with a one-step CLIA on the ADVIA Centaur^®^ cp automated chemiluminescence immunoassay system according to the manufacturer’s protocols (Siemens Healthcare Diagnostics, Camberley, Surrey, United Kingdom).

### Plasma Lipidomics

Lipid extracts were prepared using the modified Folch protocol (Folch et al., 1956). Briefly, an aliquot (100 µL) of each plasma sample was spiked with 10 µL of an isotopically-labeled lipid standard mixture (SPLASH™ Lipidomix^®^ Mass Spec Standard, Avanti Polar Lipids) before lipid extraction. Plasma lipids were extracted using chloroform, methanol (2:1), and water, dried down and reconstituted into 200 µL IPA/MeOH (1:1). To monitor system stability, a quality control (QC) sample was prepared by mixing the same aliquot from all of the samples. Chromatographic separation was accomplished using a Thermo Ultimate 3,000 system equipped with an ACQUITY UPLC^®^ BEH C18 (100 × 2.1 mm, 1.7 μm, Waters). Gradient elution of analytes was carried out with acetonitrile:water = 60:40 (0.1% formic acid + 10 mM ammonium formate) (A) and isopropanol:acetonitrile = 90:10 (0.1% formic acid + 10 mM ammonium formate) (B) at a flow rate of 0.25 ml/min. A 28-min gradient elution from 30 to 100% of solvent B was applied according to the method described by Castro-Perez et al. (2010). The electrospray ionization (ESI)-MSn experiments were performed on the Q ExactiveTM Focus mass spectrometer (ThermoFisherTM Scientific) in both positive and negative modes. A full scan by the Orbitrap analyzer comprised a mass range of 150–2000 m/z at a mass resolution of 35,000. The data dependent acquisition (DDA) method with dynamic exclusion was performed by higher energy collisional dissociation (HCD) fragmentation. Thermo Scientific™ LipidSearch™ 4.1 SP2 software was used for lipid identification and quantitation. The following search parameters were applied: product search, precursor and product mass tolerance = 5 ppm, m-Score threshold = 5. Ion adducts included H^+^, NH_4_^+^ for positive ion mode and H^−^ for negative ion mode. Lipids with grade A–C were accepted. Lipid concentrations of identified lipid species across eight major lipid classes were calculated relative to the isotopically-labeled internal standards.

### Co-occurrence Network Analysis

Weighted gene co-expression network analysis (WGCNA) (Langfelder and Horvath, 2008) was used to generate a co-occurrence network based on the Z-score transformed lipid data. Module identification was performed with the following major parameters: minModuleSize = 10 and mergeCutHeight = 0.25 (Langfelder et al., 2008). The module-trait correlations were determined by assessing the potential correlations among module eigengenes (MEs) and traits (TSH and T3/T4). Visualization of the network was performed using Cytoscape_3.8.0 (Shannon et al., 2003).

### Statistical Analysis

A non-compartmental analysis (NCA) model was used to calculate the TSH PK parameters, using WinNonlin 6.4 software. To maximize the number of differentially abundant lipid species at each time-point, the orthogonal projection to orthogonal partial least squares-discriminate analysis (OPLS-DA) model was applied using SIMCA-P software (version 14.1, Umetrics AB, Umea, Sweden). Z-scores across each of the time-point were calculated as the mean concentration of each lipid divided by the number of biological replicates. One-way analysis of variance (ANOVA) was implemented in R programming language to determine the existence of statistically significant differences across in lipids at all time-points in each of the groups. Lipids with Benjamini–Hochberg adjusted *p*-values < 0.05 were retained for further clustering analysis. The data were then clustered using a hierarchical clustering approach and separated into different clusters by applying the cutree function in R. The R “pheatmap” package was used to create a heatmap of lipid profiles. All analyses were conducted using R (3.6.0, www.r-project.org).

## Results

### Pharmacokinetic Analysis

**FIGURE 1:**
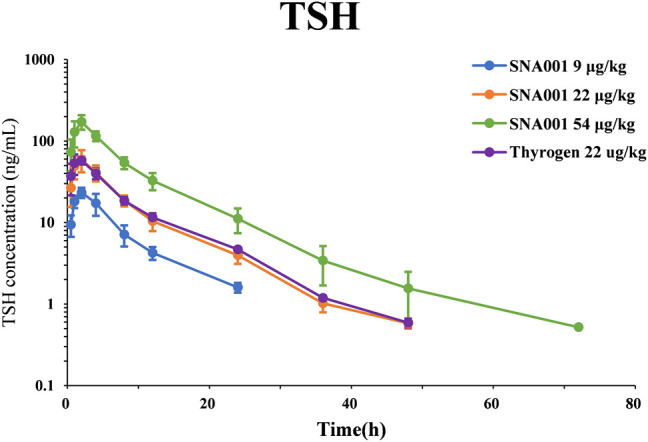
Mean (SD) observed plasma rhTSH concentration-time profile.

The PK parameters determined using TSH plasma levels following rhTSH administration in cynomolgus monkeys are presented in Table 1. Plasma TSH levels (mean ± standard deviation, SD) reached peak values within 2 h of administration (23.32 ± 3.34 ng/ml, 59.33 ± 17.85 ng/ml, 172.37 ± 34.93 ng/ml, 59.77 ± 8.17 ng/ml in the SNA001 9 μg kg^−1^, 22 μg kg^−1^, and 54 μg kg^−1^ and Thyrogen 22 μg kg^−1^ groups, respectively) and gradually decreased in concentration over time up to 48 h. The primary TSH (SNA001) PK parameters, C_max_, AUC_0–96h_, and AUC_0–inf,_ increased in a dose-dependent manner across the dose range of 9–54 μg kg^−1^. There were no significant differences in the remaining PK parameters (Tmax, T_1/2_, and MRT). Mean (±SD) plasma concentration-time profiles for TSH in each dose group are shown in Figure 1. No significant difference was found for any plasma PK parameters between the SNA001 and Thyrogen groups at the same dose. The Power model ln (y) = *β*0 + *β*1 × log(dose) was used to evaluate the proportional dose-response relationship of SNA001. Among the three groups, the β1 values for AUC_0–t_, AUC_0–inf_, and C_max_ were 1.11 [90% confidence interval (CI) 0.98–1.24], 1.16 [90% CI 1.07–1.25], and 1.11 [90% CI 1.02–1.20] respectively, indicating dose proportionality.

**TABLE 1:** Pharmacokinetic parameters (mean ± SD).

Parameters	SNA001	SNA001	SNA001	Thyrogen
	9 μg kg^−1^	22 μg kg^−1^	54 μg kg^−1^	22 μg kg^−1^
t_1/2_ (h)	7.79 ± 1.96	7.19 ± 1.13	7.86 ± 1.4	7.8 ± 0.51
T_max_ (h)	2 ± 0	2 ± 0	2 ± 0	1.67 ± 0.52
C_max_ (ng/ml)	23.32 ± 3.34	59.33 ± 17.85	172.37 ± 34.93	59.77 ± 8.17
AUC_last_ (h·μg/mL)	0.18 ± 0.03	0.48 ± 0.08	1.41 ± 0.16	0.5 ± 0.06
AUC_inf_ (h·μg/mL)	0.2 ± 0.03	0.49 ± 0.08	1.42 ± 0.17	0.51 ± 0.06
V (ml/kg)	537.97 ± 192.19	486.81 ± 136.81	431.43 ± 55.56	489.26 ± 72.13
Cl (ml/h/kg)	46.76 ± 6.05	46.34 ± 7.48	38.48 ± 4.6	43.39 ± 4.96
MRT (h)	6.56 ± 0.24	8.13 ± 1.25	8.72 ± 1.7	8.71 ± 0.64
AUC_(0–24h)_ (h·μg/mL)	0.18 ± 0.03	0.44 ± 0.09	1.28 ± 0.13	0.46 ± 0.06

T_1/2_ (h), elimination half-life; T_max_ (h), time to maximum plasma concentration; C_max_ (ng/ml), maximum plasma concentration; AUC_last_ (h·μg/mL), area under the concentration-time curve from time zero to last quantifiable concentration; AUC_inf_, area under the concentration-time curve from time zero to infinity; Cl, Clearance; MRT (h), mean residence time.

### Pharmacodynamic Study of T3/T4 Concentrations

**FIGURE 2:**
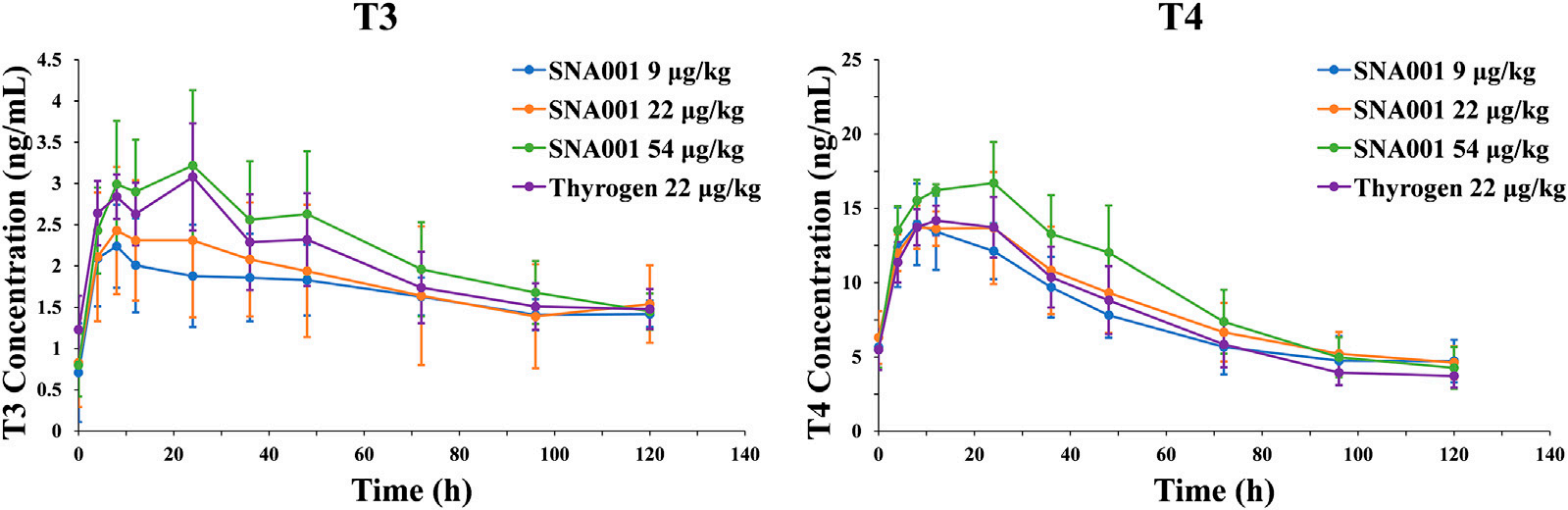
Mean (SD) observed T3/T4 concentration-time profile. Left, plasma T3 concentrations. Right, plasma T4 concentrations.

Serum T3/T4 concentrations increased rapidly after administration of rhTSH (Figure 2). Maximal serum T3/T4 concentrations were reached within 24 h. Comparative analysis revealed that the peak serum T3 and T4 concentrations (mean ± SD) after 54 μg kg^−1^ SNA001 administration were slightly higher than those after 22 μg kg^−1^ SNA001 administration (T3: 3.22 ± 0.91 μg/ml vs. 2.43 ± 0.77 μg/ml, *p* = 0.136; T4: 13.72 ± 1.46 μg/dl vs. 16.7 ± 2.76 μg/dl, *p* = 0.041). There were no significant differences in the peak concentrations of T3 and T4 following administration of 22 μg kg^−1^ SNA001 and 22 μg kg^−1^ Thyrogen (T3: *p* = 0.145; T4: *p* = 0.533). Spearman’s rank correlation analysis was also performed to evaluate the relationships between TSH and T3/T4 concentrations. TSH concentration showed a significant positive correlation with T3/T4 (*r* > 0.6, *p* < 0.05) in all rhTSH dose groups (Supplementary Table S1).

### A Global Analysis of rhTSH-Stimulated Plasma Lipids

To investigate the time-course profile of lipid concentrations stimulated by different rhTSH doses, total lipids from 144 plasma samples were extracted and analyzed by UPLC-HR-MS. The peak areas of the individual lipid species were normalized against the peak area of the internal standard (IS) spiked into each sample (Supplementary Table S2). Normalized lipid values were averaged for each time-point across all individuals (*n* = 6). In total, 420 lipids were detected and quantified in all samples at all time-points. These lipid molecules included phospholipids (196 molecules), sphingolipids (60 molecules), and neutral lipids (164 molecules). Of the major phospholipid classes, we identified 117 phosphatidylcholines (PCs), 39 lysophosphatidylcholines (LPCs), and 33 phosphatidylethanolamines (PEs). The triacylglycerols (TGs) were the largest class of lipids identified (152 molecules).

**FIGURE 3:**
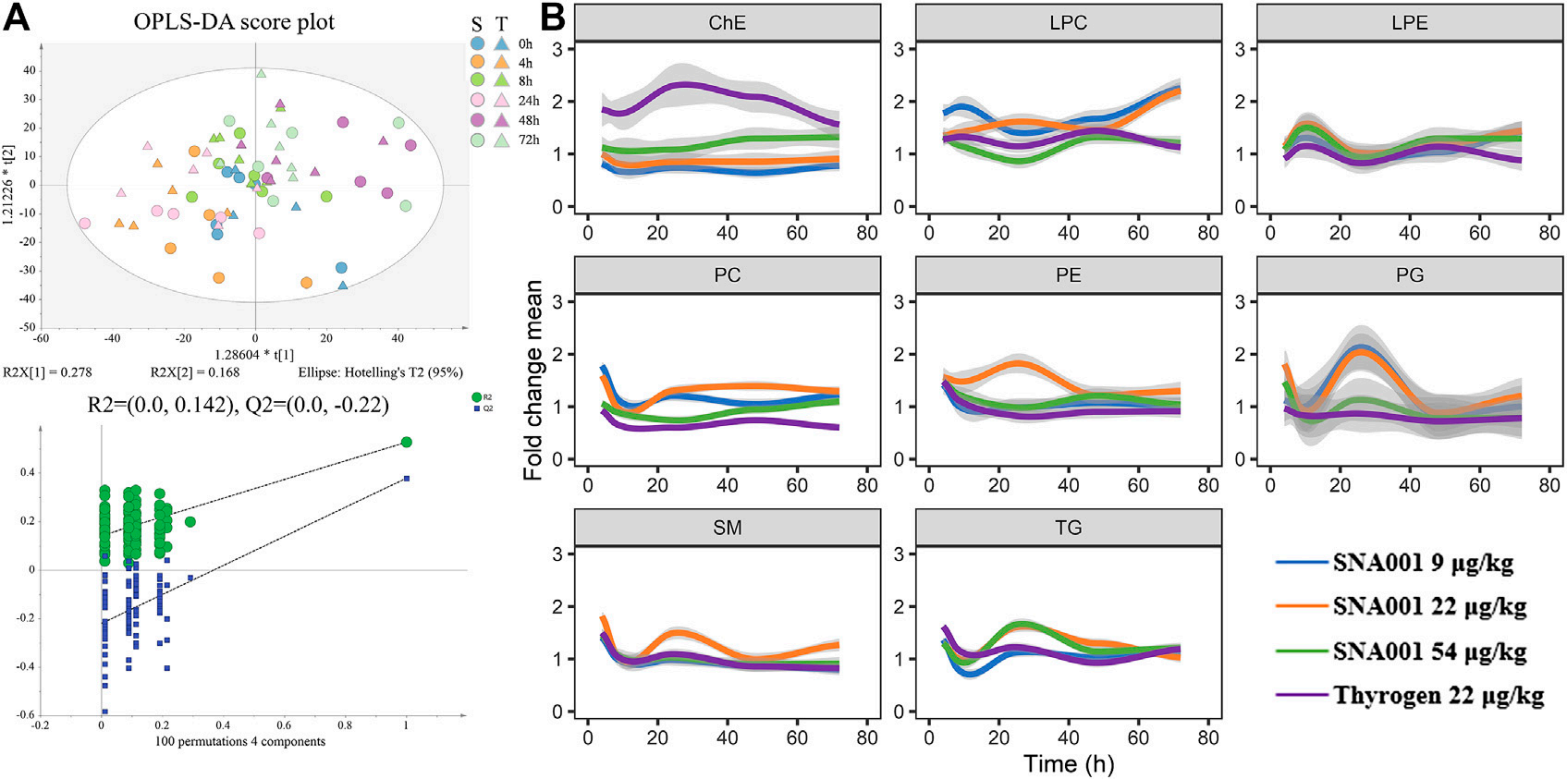
Characterization of the rhTSH-stimulated plasma lipidomics. **(A)** Upper, OPLS-DA score plot of the plasma lipidomics from the 22 μg kg^−1^ SNA001(S) and Thyrogen(T) groups. Lower, the OPLS-DA model was well-validated by a permutation test (*n* = 100). **(B)** Time series profiles of lipid species clustered by lipid class. Values were averaged across all individuals (*n* = 6) for each time-point. The thick colored line represents the average of each lipid class.

Multivariate OPLS-DA was used to assess metabolomic changes in the 22 μg kg^−1^ SNA001 and Thyrogen groups. As shown in Figure 3A, the cumulative R^2^Y was 0.328 and Q^2^ was 0.146. Changes in lipid levels at each point were obvious, and samples in SNA001 and in Thyrogen groups were comparable in the OPLS-DA score plot. The profile changes, especially those at 4 h and 24 h, were also detected in all dose groups (Supplementary Figure S1). Next, all lipid profiles were clustered according to lipid class (Figure 3B). A clear pattern of elevated TG and SM levels was observed at earlier time-points (4 h), with subsequent increases peaking at approximately 24 h. In contrast, PE levels peaked at a later at 8 h and 48 h after rhTSH administration in all dosage groups. Variable changes in the levels of lipids in other classes were observed. Furthermore, no significant changes of plasma lipid were observed in control group without rhTSH administration in 24 h (Supplementary Figure S2).

### Metabolic Clustering of Lipid species

Next, we investigated unique lipid changes in response to the route of rhTSH administration. Of 420 analyzed lipid species, the levels of 224 were significantly changed in at least one rhTSH group during 72 h after administration (ANOVA 0.049 ≥ *p* ≥ 1.19 × 10^–12^, Supplementary Table S3). Because biologically related lipid groups may exhibit similar expression patterns, we analyzed the expression pattern of rhTSH stimulated lipid species by hierarchical clustering and the dendrogram was divided into five clusters using the cutree function in R (Supplementary Figure S3A). Cluster 4 (C4) and cluster 3 (C3) contained the highest numbers of lipids (64 and 61, respectively), with lipid profiles of C4 peaked at 4 h and C3 peaked at 24 h after administration. These were followed by cluster 5 (C5), with the highest concentration at 4 h after rhTSH administration, and cluster 2 (C2), which had sharply increased levels of 27 lipids at 72 h. The final cluster, C1, contained increased levels of 19 lipids with peaks at 8 h (Supplementary Figure S3B and Supplementary Table S4).

SM (d42:1) and SM (d44:2) were the most representative lipids in cluster four that showed significantly changed levels at 4 h and 24 h (*P*: 3.02e−06; *P*: 7.11e−06 for SNA001 22 μg kg^−1^). Thirty long-chain TGs (TG52–TG60) and 23 SMs lipids accounting for 82.8% of lipids in cluster four showed a higher degree of alignment, which is consistent with the individual profile analysis of TGs and SMs. The changes in lipid levels in cluster four were comparable for all the doses of SNA001 (9, 22, and 54 μg kg^−1^) and Thyrogen (22 μg kg^−1^). In cluster 2, the levels of 64.2% of shorter chain TG (TG32–TG47) lipids changed dramatically at 72 h compared to other time-points as the dose of SNA001 increased (in dose-dependent manner) (9, 22, and 54 μg kg^−1^).

### Lipid Co-occurrence Network and TSH and T3/T4 Associations

To identify relationships between TSH and T3/T4 and their potential combined effect on plasma lipidomic output, we constructed a network of co-occurrence lipid species and interrogated the network for modules using WGCNA. Seven distinct lipid modules containing between 13 and 160 lipids in each module were detected in the SNA001 dose group (9 μg kg^−1^) (Supplementary Figure S4A). The complete list of lipid species and their module organization is listed in Supplementary Table S5. Lipid co-occurrence network analysis revealed that lipids from the same lipid class clustered into the same modules preferentially (Supplementary Figures S4B,C).

The module eigengene (ME) is the first principal component of a given module and can be considered as a representative of the module’s lipid profile. To explore meaningful modules associated with traits, module-trait associations of MEs with TSH and T3/T4 were analyzed. It was found that the MEturquoise module for SNA001 dose (9 μg kg^−1^), containing lipids derived from PC, PE, SM, and TG, had the strongest association with TSH (*r* = 0.88, *p* = 0.02) and were also positively correlated with T3/T4 (T3: *r* = 0.45; T4: *r* = 0.48). The MEred module, representing lipids derived mainly from LC, and LPC, was also highly positively correlated with T3, whereas the MEblack module was negatively associated with T3/T4 (Supplementary Figure S4D). We also constructed a lipid co-occurrence network for the remaining rhTSH dose groups. Our data showed modules, especially MEturquoise and MEblue, containing lipids derived mainly from PC, PE, SM, and TG, were preserved in all networks (Supplementary Figure S5A). Furthermore, the MEturquoise module was positively correlated with TSH in all the dose groups (Supplementary Figure S5B).

## Discussion

This study provides the first systematic analysis of thyroid hormones changes and lipid changes in cynomolgus monkeys. Taking full advantage of UPLC-HR-MS lipidomic technology, we have illustrated the landscape of the dynamic lipid changes stimulated by rhTSH. Our findings indicate a potential role for TSH in the induction of T3/T4 alterations in lipid profiles and provide an insight into the mechanism underlying the role of thyroid hormone in metabolic syndrome.

In the current study, T3 and T4 concentrations peaked approximately 24 h after administration of exogenous rhTSH, which is 20 h later than the peak TSH concentration. Fluctuations in T3 and T4 concentrations were also observed in the first 24 h after rhTSH administration. Clinical studies of the circadian rhythms of endogenous thyroid hormones showed that changes in FT3 concentrations exhibit a periodicity that lags behind those of TSH (Russell et al., 2008). The mechanism was also confirmed by a comprehensive mathematical modeling of the pituitary-thyroid feedback loop (Berberich et al., 2018). Furthermore, Torres et al., described that the peak serum T3/T4 concentration was delayed by approximately 24 h–48 h after exogenous rhTSH injection in normal healthy volunteers (Ramirez et al., 1997; Torres et al., 2001). The mechanism responsible for the delay in thyroid stimulation by rhTSH has also been demonstrated in the rhesus monkey (Braverman et al., 1992). Therefore, it seems likely that the mechanism of circadian rhythm and biological action of TSH and thyroid hormones contribute to the perturbed T3/T4 concentration stimulated by rhTSH in the present study.

The effects of TSH on thyroid hormone (TH) metabolism and concentrations of plasma lipids are still not clear. Various reports have illustrated the mechanism by which lipid metabolism is induced directly by TSH (Yan et al., 2014; Gong et al., 2017) or by thyroid hormones (Prieur et al., 2005; Masson et al., 2009). Recently, a retrospective clinical study of 82 thyroidectomized patients with differentiated thyroid cancer (DTC) showed that TG increased significantly, while serum HDL-cholesterol and LDL-cholesterol decreased slightly after rhTSH stimulation (Beukhof et al., 2018). Similar results showing that TSH has a direct effect on total cholesterol and TGs were also obtained from a recent study of 27 premenopausal women with DTC after rhTSH administration (Delitala et al., 2020). Although the total thyroidectomy DTC model is an ideal for analyzing the direct effect of TSH alone on serum lipids in which FT4 values remain stable, it is still unclear whether the rise in lipid levels is mediated by TSH alone or in combination with thyroid hormones. In this study, we showed a significant increase in serum lipid concentrations after rhTSH administration. We found that the pattern of changes in the levels of lipids such as TG and SM correlated with the pattern of changes in rhTSH and T3/T4 concentrations, which seems to support a combined effect of TSH and T3/T4 stimulation. Furthermore, module-trait association analyses between lipid modules and TSH and T3/T4 indicated that TSH is the dominant driving factor in stimulating global changes in lipid profiles of cynomolgus monkeys.

One of strengths of this study is the indication that even low SNA001 dose (9 μg kg^−1^) stimulated significant changes in T3/T4 and lipids, and the stimulated profile was comparable to that induced by Thyrogen. Such nonclinical studies will be used to support the optimal SNA001 dose in two ongoing clinical trials in DTC patients (CTR20182349, CTR20192559) in China. Limitations of our study include the untargeted lipidome included in this study and the lack of absolute quantification of lipidomic features, which may reduce the accuracy of the statistical analysis. A second limitation was the lack of illustration of molecular evidence and functions of specific lipid species in blood for the relationship between TSH, thyroid hormones and lipids, which requires further investigations in cells and other animal models.

## Conclusion

The present study is the first to show the effects of administration of exogenous rhTSH in cynomolgus monkeys, which in turn modulates plasma lipid concentrations toward a more unfavorable profile. The plasma lipidome and changes in lipid levels were associated with TSH and T3/T4 concentrations. Furthermore, we also demonstrated that the T3/T4 effects on the lipid profile were delayed after TSH stimulation. Further studies are required to confirm our findings and clarify the molecular mechanisms by which TSH affects peripheral TH metabolism and lipid profiles.

## Data Availability

The original contributions presented in the study are included in the article/Supplementary Material, further inquiries can be directed to the corresponding authors.
