# Supplementary material for: Lipidomic Perturbations in Cynomolgus Monkeys are Regulated by Thyroid Stimulating Hormone
Source: Front Mol Biosci. 2021 Mar 15;8:640387. doi: 10.3389/fmolb.2021.640387 (PMC8006939; doi:10.3389/fmolb.2021.640387)
Supplement: Supplementary file 1 [file datasheet1.docx]

Supplementary Material

# Supplementary Figures and Tables

## Supplementary Figures

**Supplementary Figure S1.** OPLS-DA score plot and validation plot of the plasma lipidomics from the SNA001 9 μg kg^-1^ (A), SNA001 22 μg kg^-1^ (B), 54 μg kg^-1^ (C), and thyrogen 22μg kg^-1^ (D) groups.


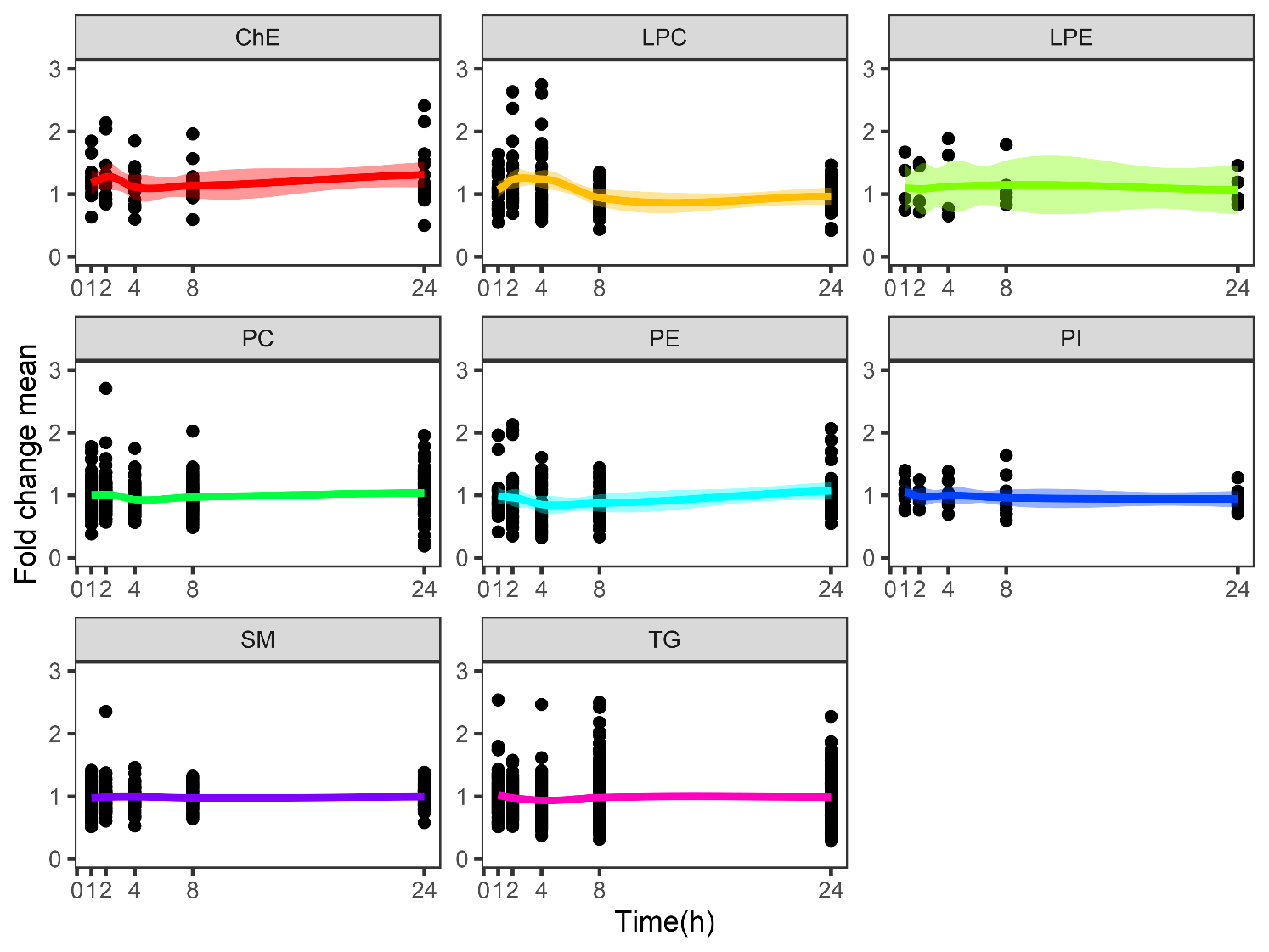


**Supplementary Figure S2.** Time series profiles of lipid species clustered by lipid class from controls during a 24h period. Values were averaged across all individuals (n = 6) for each time-point. The thick colored line represents the average of each lipid class.


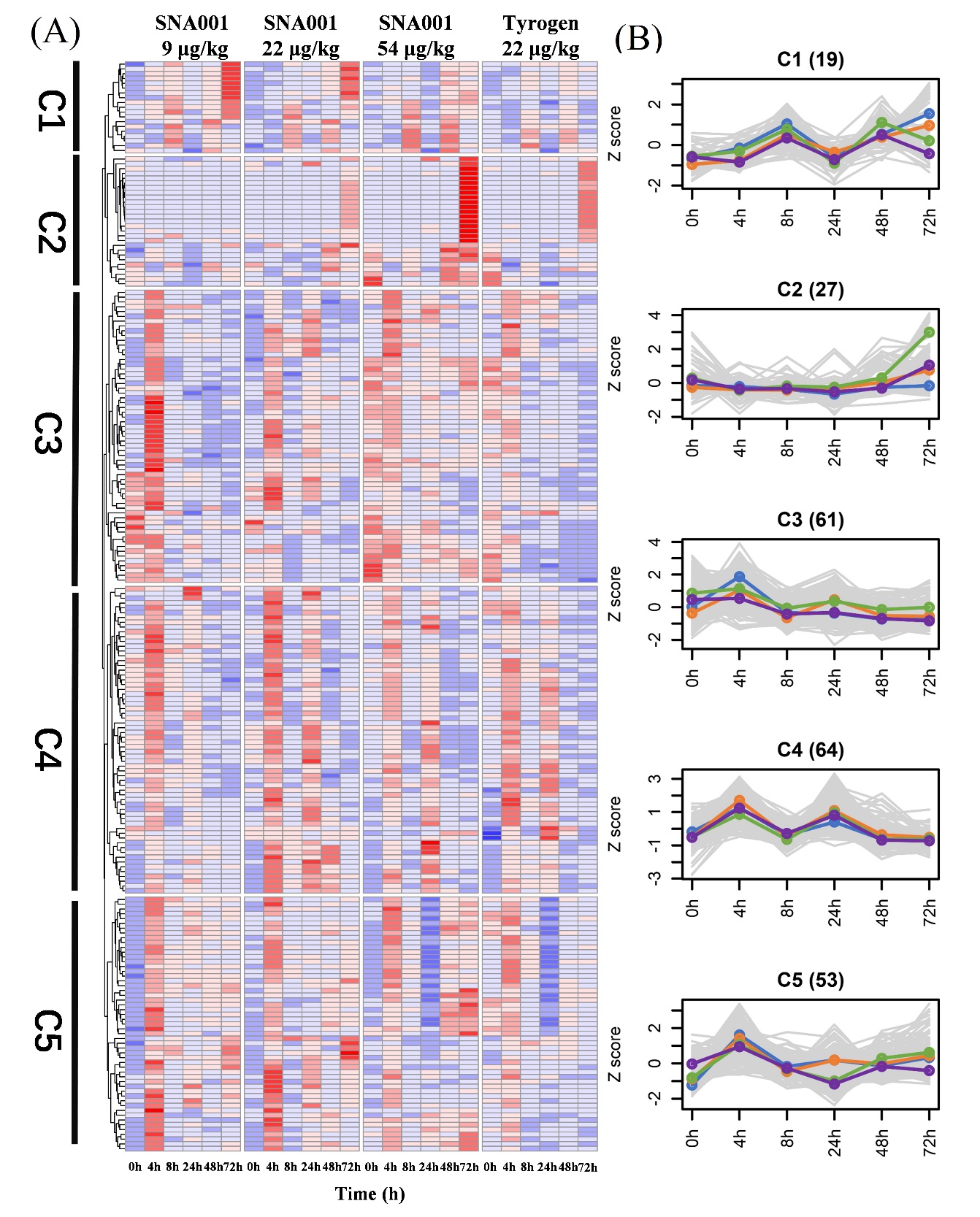


**Supplementary Figure S3.** Time-dependent transient changes in lipid profiles. (A) Hierarchical clustering of differential lipid species according to ANOVA. (B) Identification of five lipid cluster profiles with coherent changes according to hierarchical clustering. The thick colored dotted line represents the average of each lipid cluster in each dose group (blue: SNA001 9 μg kg^-1^; orange: SNA001 22 μg kg^-1^; green: SNA001 54 μg kg^-1^; purple: thyrogen 22 μg kg^-1^).


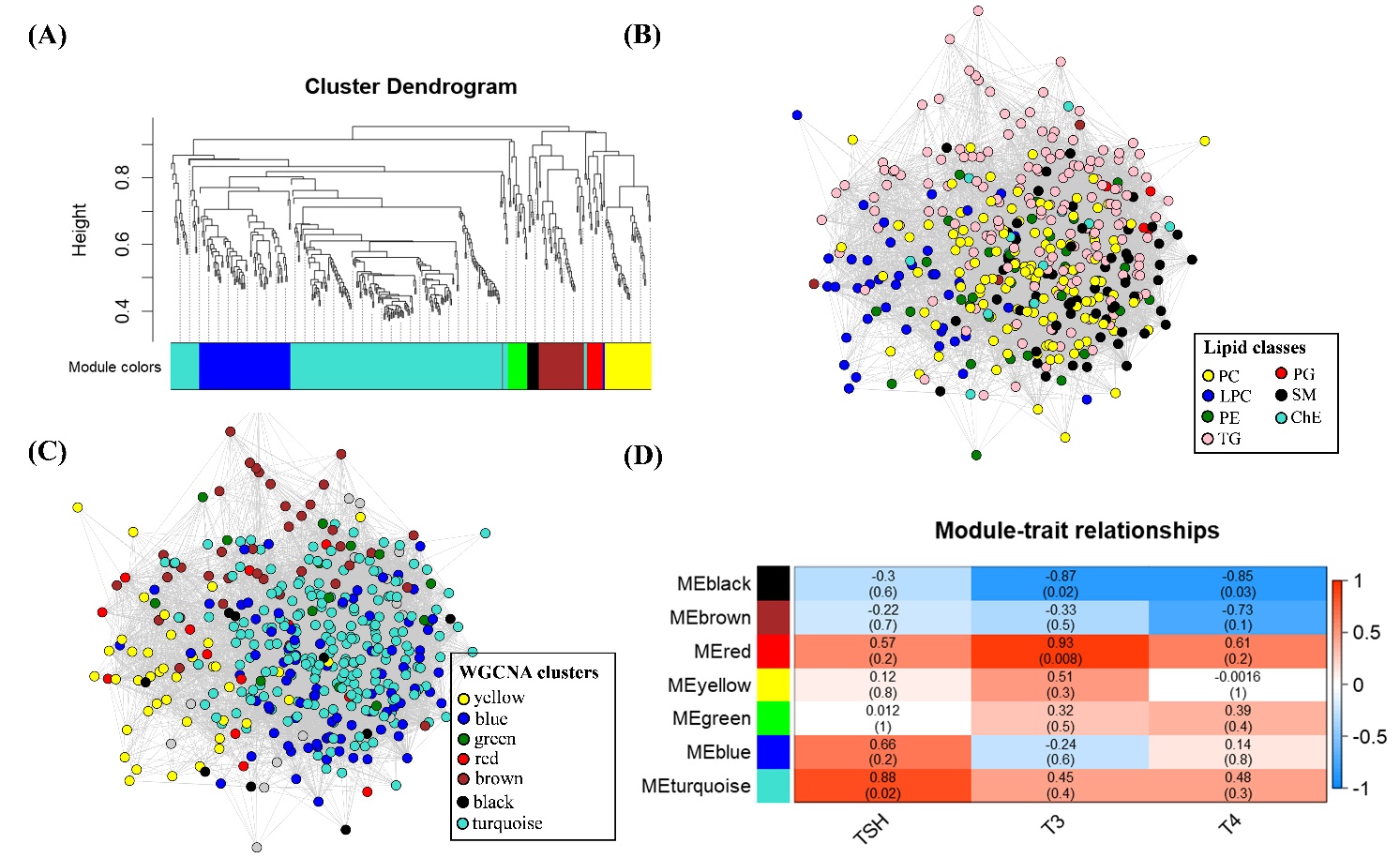


**Supplementary Figure S4.** Lipid co-occurrence network and module-trait associations of the SNA001 9 μg kg^-1^ group. (A) Hierarchical clustering dendrogram of lipid species, together with assigned module colors from the WGCNA. Each vertical line corresponds to one lipid. (B) Lipid co-occurrence network where lipids (nodes) are colored according to the lipid classes to which they belong. Edges corresponded to Pearson correlations with *P*-values ≤ 0.01 and abs(*r*) ≥ 0.7 in all rhTSH dose groups retained. (C) Lipid co-occurrence network where lipids (nodes) are colored according to the WGCNA module colors. (D) Module–trait associations are presented. Each cell of the matrix contains the correlation and the corresponding *P-*value. The table is color-coded by correlation (red, positive correlation; blue, negative correlation).


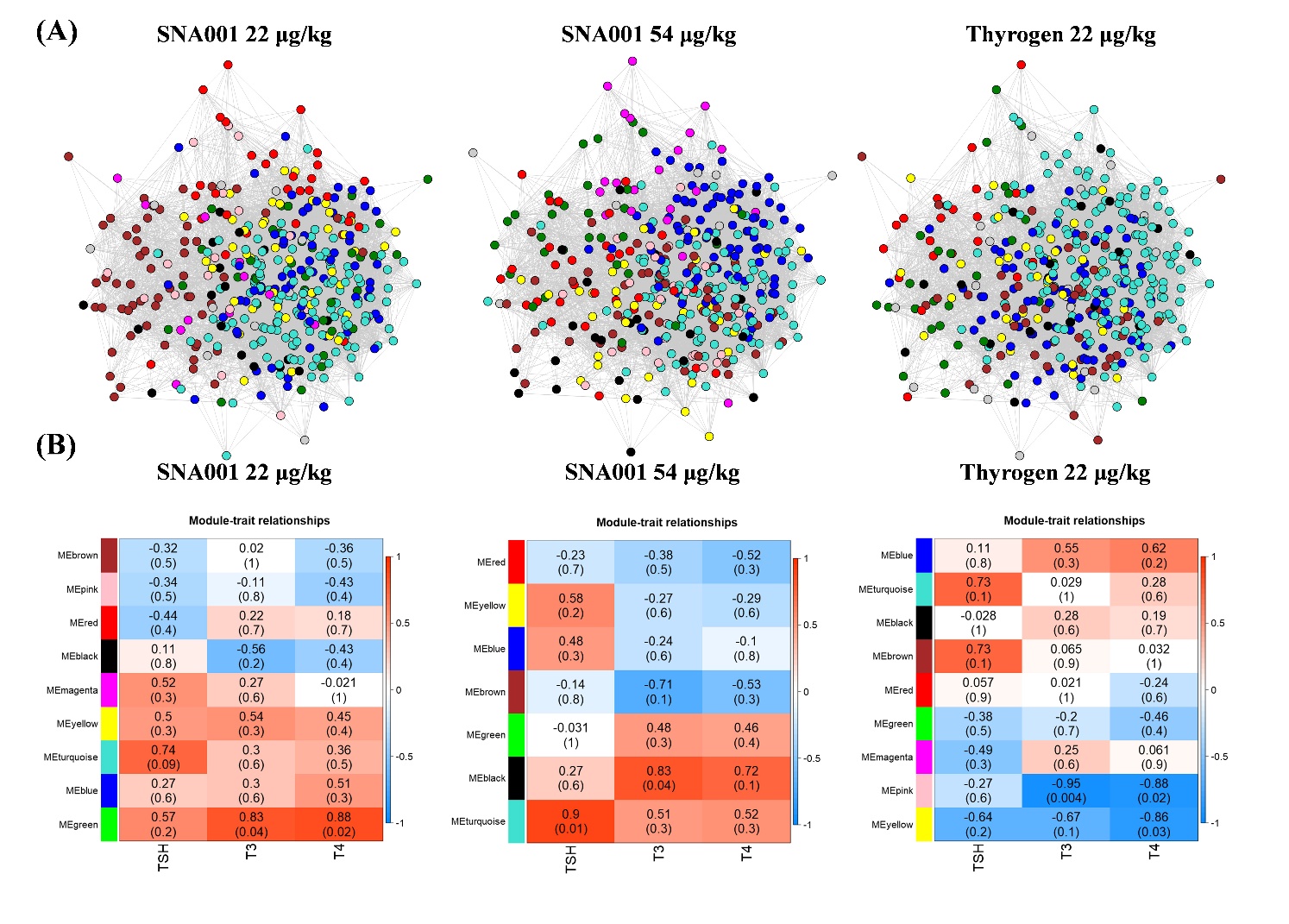


**Supplementary Figure S5.** Comparison of rhTSH lipid networks. (A) Lipid co-occurrence networks for SNA001 (22 and 54 μg kg^-1^) and thyrogen (22 μg kg^-1^). Lipids (nodes) are colored according to their corresponding WGCNA module colors. Edges are represented only for significant correlations (*P-*value ≤ 0.01, abs (*r*) ≥ 0.7). (B) Relationships of module eigengenes and TSH, T3/T4 in the SNA001 (22 and 54 μg kg^-1^) and thyrogen (22 μg kg^-1^) data. Rows and columns in the table correspond to lipid modules and traits, respectively. Each cell of the matrix contains the correlation and the corresponding *P-*value. The table is color-coded by correlation according to the color legend.

## Supplementary Tables

**Table S1.**Spearman’s rank correlation between TSH and T3/T4.**Table S2.**Lipid concentrations from 144 plasma samples.**Table S3.**Significantly differential lipid species altered in all time-points using ANOVA analysis**. Table S4.** Identification of 5 lipid cluster profiles by hierarchical clustering.**Table S5.** The complete list of lipid species and their WGCNA modules for all rhTSH dose groups.
